# Structure of the substrate-binding domain of *Plasmodium falciparum* heat-shock protein 70-x

**DOI:** 10.1107/S2053230X2001208X

**Published:** 2020-09-28

**Authors:** Julia Schmidt, Ioannis Vakonakis

**Affiliations:** a Department of Biochemistry, University of Oxford, South Parks Road, Oxford OX1 3QU, United Kingdom

**Keywords:** malaria, chaperones, *Plasmodium falciparum*, erythrocyte remodelling, PfHsp70-x, PfEMP-1, crystallography, complexes

## Abstract

The malaria parasite *Plasmodium falciparum* extensively modifies erythrocytes that it invades by exporting a large complement of proteins to the host cell. Among these exported components is a single heat-shock 70 kDa class protein, PfHsp70-x, that supports the virulence and growth rate of the parasite during febrile episodes. The ATP-binding domain of PfHsp70-x has previously been resolved and showed the presence of potentially druggable epitopes that differ from those on human Hsp70 chaperones. Here, the crystallographic structure of the substrate-binding domain (SBD) of PfHsp70-x is presented in complex with a hydrophobic peptide. The PfHsp70-x SBD is shown to be highly similar to the counterpart from a human erythrocytic Hsp70 chaperone. The binding of substrate at the interface between β-sandwich and α-helical subdomains of this chaperone segment is also conserved between the malaria parasite and humans. It is hypothesized that the parasite may partly exploit human chaperones for intra-erythrocytic trafficking and maintenance of its exported proteome.

## Abstract 2

The 3.25 Å resolution crystallographic structure of the substrate-binding domain of the erythrocyte-exported Hsp70 chaperone from the malaria parasite *Plasmodium falciparum* was determined. The parasite domain is shown to be highly similar to the equivalent domain of a human Hsp70 chaperone in structure and mode of substrate engagement.

## 1. Introduction   

Malaria is an acute febrile illness caused by five different *Plasmodium* species in humans (World Health Organization, 2019 ▸). Of these, *P. falciparum* is the most prevalent and dangerous species, accounting for nearly 95% of malaria deaths globally. Key to *P. falciparum* virulence is the induction of strong cell adhesion in the erythrocytes that it invades, which is a characteristic of this parasite species among human-infective *Plasmodia* (Craig *et al.*, 2012 ▸; Smith *et al.*, 2013 ▸). The adhesion of parasitized erythrocytes to endothelial cells, such as those in the microvasculature lining of the brain, kidneys or placenta, and to other erythrocytes, forming cell clumps, leads to blood-vessel blockage, tissue damage from oxygen deprivation and inflammation, and potentially to coma and death.

Erythrocyte cytoadherence is the result of extensive host-cell remodelling mediated by parasite proteins exported to the host, collectively referred to as the parasite ‘exportome’ (Spillman *et al.*, 2015 ▸). The exported proteins include the *P. falciparum* erythrocyte membrane protein 1 (PfEMP1) family of adhesion ligands that mediate cell attachment (Hviid & Jensen, 2015 ▸), members of the *Plasmodium* helical interspersed sub-telomeric (PHIST) protein family that act as interaction hubs (Warncke *et al.*, 2016 ▸), and parasite components that remodel the host membrane and cytoskeleton, create nutrient-permeability pathways and contribute to immune-system evasion (de Koning-Ward *et al.*, 2016 ▸; Proellocks *et al.*, 2016 ▸; Goldberg & Cowman, 2010 ▸). *P. falciparum* also exports a single heat-shock 70 kDa-class protein chaperone, PfHsp70-x (PF3D7_0831700; Külzer *et al.*, 2012 ▸), to the host cell. Hsp70-class chaperones play key roles across the kingdoms of life in protein quality control, protein translocation, folding and assembly of complexes, and preventing protein aggregation (Boorstein *et al.*, 1994 ▸; Meimaridou *et al.*, 2009 ▸). PfHsp70-x is found in the parasitophorous vacuole, where it may associate with the PTEX translocation machinery (Zhang *et al.*, 2017 ▸), as well as in the host-cell cytoplasm, where it localizes in mobile structures, the ‘J-dots’, together with stimulatory Hsp40-class co-chaperones and the PfEMP1 adhesion ligand (Külzer *et al.*, 2012 ▸). Thus, it is thought that PfHsp70-x assists in PfEMP1 folding and in the assembly of a virulence complex on the erythrocyte membrane that includes PfEMP1. Consistent with this analysis, parasite lines lacking PfHsp70-x exported PfEMP1 less efficiently and were ∼60% less adherent compared with control parasites (Charnaud *et al.*, 2017 ▸). Furthermore, in addition to its role in parasite virulence, PfHsp70-x supports cell viability at elevated temperatures, as PfHsp70-x depletion reduced parasite growth by ∼40% during heat shocks comparable to the febrile episodes of malaria patients (Day *et al.*, 2019 ▸). These studies have established PfHsp70-x as a key component for parasite survival and virulence.

The function of Hsp70-class chaperones is linked to the binding of exposed hydrophobic segments of aggregation-prone proteins, ‘holding’ these and facilitating their correct folding in a catalytic cycle driven by ATP hydrolysis (Boorstein *et al.*, 1994 ▸; Meimaridou *et al.*, 2009 ▸). The Hsp70 structure comprises an N-terminal nucleotide-binding domain (NBD) and a C-terminal substrate-binding domain (SBD) subdivided into twisted β-sandwich (SBDβ) and α-helical lid (SBDα) parts; the NBD and SBD temporarily associate as part of the catalytic cycle, which also includes the opening and closing of the SBDβ/SBDα subdomains to trap the unfolded substrate at their interface. We previously resolved the crystallographic structure of the PfHsp70-x NBD as well as that of the cognate parasite co-chaperone PFA0660w (Day *et al.*, 2019 ▸), which stimulates PfHsp70-x activity (Daniyan *et al.*, 2016 ▸), and used these to create a hybrid model of the chaperone–co-chaperone complex that could be compared with human Hsp70 chaperone structures. Here, we report the PfHsp70-x SBD structure in the domain-closed state complexed with a model substrate peptide, used in previous studies of the human Hsp70 and *Escherichia coli* DnaK SBDs (Zhang *et al.*, 2014 ▸; Zhu *et al.*, 1996 ▸), that emulates exposed hydrophobic segments of unstructured or misfolded proteins. We show that the PfHsp70-x SBD is highly similar to the human erythrocytic chaperone Hsp70 in structure and mode of substrate binding.

## 2. Materials and methods   

### 2.1. Macromolecule production   

A synthetic, codon-optimized DNA fragment (Integrated DNA Technologies) encoding PfHsp70-x residues 424–650 (PfHsp70-x SDB; UniProt K7NTP5) was cloned in the pFLOAT vector (Rogala *et al.*, 2015 ▸), which provides an N-terminal His_6_ tag separated from the target protein by a Human rhinovirus (HRV) 3C protease cleavage site (Table 1 ▸). The plasmid construct was transformed into *Escherichia coli* Rosetta 2(DE3) competent cells (Novagen). The transformed *E. coli* cells were used to inoculate lysogeny broth medium supplemented with appropriate antibiotics and the cells were grown at 37°C to an OD_600_ of ∼0.6. Protein expression was induced by the addition of a 0.25 m*M* final concentration of isopropyl β-d-thiogalactopyranoside and was left to proceed at 18°C overnight. The cells were harvested by centrifugation and were resuspended in lysis buffer [500 m*M* NaCl, 20 m*M* Na_2_HPO_4_ pH 7.2, 0.01%(*v*/*v*) Triton X-100]. The cells were lysed by sonication and the lysates were clarified by centrifugation for 60 min at 25 000*g*.

The clarified cell supernatants were loaded onto metal-affinity HiTrap Talon columns (GE Healthcare Life Sciences) equilibrated in lysis buffer. The columns were washed using lysis buffer supplemented with 10 m*M* imidazole and the proteins were eluted using a gradient of imidazole concentration to 500 m*M*. Protein-containing fractions were pooled and dialysed against 3C cleavage buffer [50 m*M* NaCl, 20 m*M* Na_2_HPO_4_ pH 7.2, 1 m*M* dithiothreitol (DTT)] prior to overnight incubation with recombinant HRV 3C protease engineered with a noncleavable His_6_ tag. The HRV 3C protease was removed by a reverse metal-affinity step using a HiTrap Talon column, with PfHsp70-x SBD in the column flowthrough. The PfHsp70-x SBD samples were then loaded onto an ion-exchange HiTrap Q column (GE Healthcare Life Sciences) equilibrated in 3C cleavage buffer and eluted using a NaCl gradient to a concentration of 500 m*M*. Protein samples were concentrated by centrifugal ultrafiltration using Amicon Ultra 10 kDa cutoff devices (Merck Millipore) and were loaded onto a Superdex 75 16/600 HiLoad column (GE Healthcare Life Sciences) equilibrated in crystallization buffer [150 m*M* NaCl, 20 m*M* 4-(2-hydroxyethyl)-1-piperazine­ethanesulfonic acid (HEPES) pH 7.2, 1 m*M* DTT]. The protein-containing samples were pooled and concentrated as described above.

The protein quality was analysed by SDS–PAGE. The protein concentration was estimated by UV absorption at 280 nm using a Nanodrop spectrophotometer (Thermo Scientific) and a modified extinction coefficient calculated from the expected amino-acid sequence (Pace *et al.*, 1995 ▸). All chemicals were purchased from Sigma–Aldrich unless otherwise noted.

### 2.2. Crystallization   

A synthetically produced heptapeptide with the sequence N_1_RLLLTG_7_ (GL Biochem, Shanghai, People’s Republic of China) was dissolved directly in crystallization buffer. Crystallization samples formulated in the same buffer were composed of PfHsp70-x SBD and this peptide at final concentrations of 10 mg ml^−1^ and 5 m*M*, respectively. Samples were incubated for 15 min at 42°C, gradually cooled to room temperature and centrifuged at 15 000*g* for 5 min prior to crystallization-drop setup using the sitting-drop vapour-diffusion method. Crystallization drops of 200 nl final volume were set up using a Mosquito robot (TTP Labtech) and a 1:1 ratio of protein-containing solution to mother liquor (Table 2 ▸). Diffracting crystals developed after incubation at 20°C for three days with a mother liquor consisting of 0.1 *M* bicine/2-­amino-2-(hydroxymethyl)propane-1,3-diol (Trizma) base pH 8.5, 10%(*w*/*v*) polyethylene glycol (PEG) 20 000, 20%(*w*/*v*) PEG MME 550 and 0.03 *M* each of an ethylene glycol mixture (diethylene glycol to pentaethylene glycol).

### 2.3. Data collection and processing   

Diffraction data were collected on beamline I03 at Diamond Light Source, Harwell, UK to a resolution of 3.25 Å. Diffraction data were processed with *xia*2 in *DIALS* (Winter, 2010 ▸; Winter *et al.*, 2018 ▸). The space group was determined to be *P*12_1_1, with four copies per asymmetric unit (data statistics are given in Table 3 ▸).

### 2.4. Structure solution, refinement and analysis   

The structure was solved by molecular replacement using *Phaser* (McCoy *et al.*, 2007 ▸) with a single copy of the human Hsp70 SBD (PDB entry 4po2; Zhang *et al.*, 2014 ▸) from which the substrate peptide had been removed as a molecular-replacement model. Iterative cycles of refinement and model building were performed using *BUSTER* (Bricogne *et al.*, 2017 ▸) and *Coot* (Emsley *et al.*, 2010 ▸), respectively (statistics are given in Table 4 ▸). Figures were prepared using *PyMOL* (DeLano, 2002 ▸). Analysis of complex interfaces was performed by *PDBePISA* (Krissinel & Henrick, 2007 ▸). Sequence alignments were performed by *Clustal Omega* (Madeira *et al.*, 2019 ▸).

## 3. Results and discussion   

### 3.1. Structure of the PfHsp70-x SBD–substrate complex   

**Figure 1:**
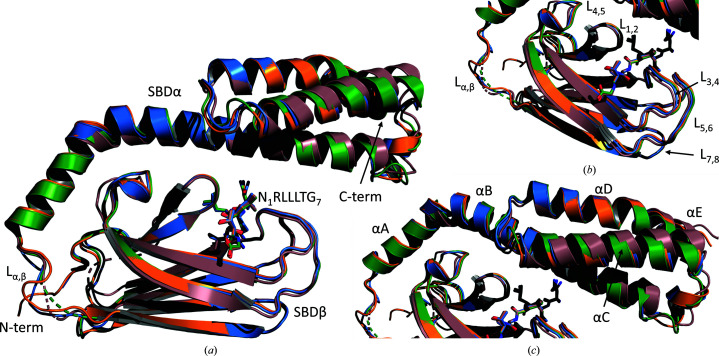
Structure of the PfHsp70-x SBD–substrate complex. (*a*) Superposition of the four complex copies in the asymmetric unit of the crystal, showing the protein architecture comprising SBDβ and SBDα subdomains. The substrate peptide (N_1_RLLLTG_7_) is shown as sticks. (*b*, *c*) Close-up views of the SBDβ and SBDα subdomains, respectively. SBDβ β-strand-connecting loops and SBDα helices are enumerated.

The asymmetric unit of the PfHsp70-x SBD–substrate crystals comprised four copies of the complex, with each protomer adopting the characteristic SBDβ/SBDα subdomain architecture of Hsp70 SBDs (Fig. 1 ▸ *a*). PfHsp70-x SBDβ (residues 424–539) is made up of eight β-strands arranged into two β-sheets of four antiparallel strands, and is connected by a short loop, L_α,β_, to SBDα (residues 540–644). SBDα consists of five α-helices, which first cape SBDβ and are then arranged into a C-terminal antiparallel three-helical bundle. In this manner, SBDα forms a helical lid across SBDβ, thereby potentially regulating access to the substrate-binding site (see below). The contact between the SBDβ and SBDα subdomains showed that our crystallographic structure corresponds to the closed state of this substrate-binding module. The four PfHsp70-x SBD monomers in the asymmetric unit were structurally similar, with an average pairwise C^α^ root-mean-square deviation (r.m.s.d.) of 2.19 Å (215 aligned atoms with no outlier rejections; Fig. 1 ▸ *a*); however, the similarity was significantly more pronounced within the SBD subdomains (SBDβ, 0.76 Å average pairwise C^α^ r.m.s.d. for 105 aligned atoms, Fig. 1 ▸ *b*; SBDα, 1.12 Å average pairwise C^α^ r.m.s.d. for 104 aligned atoms, Fig. 1 ▸ *c*). This suggested that the greatest plasticity of PfHsp70-x SBD manifests in the relative positioning of SBDβ and SBDα, which is consistent with these two subdomains being mobile relative to each other as part of the chaperone catalytic cycle.

**Figure 2:**
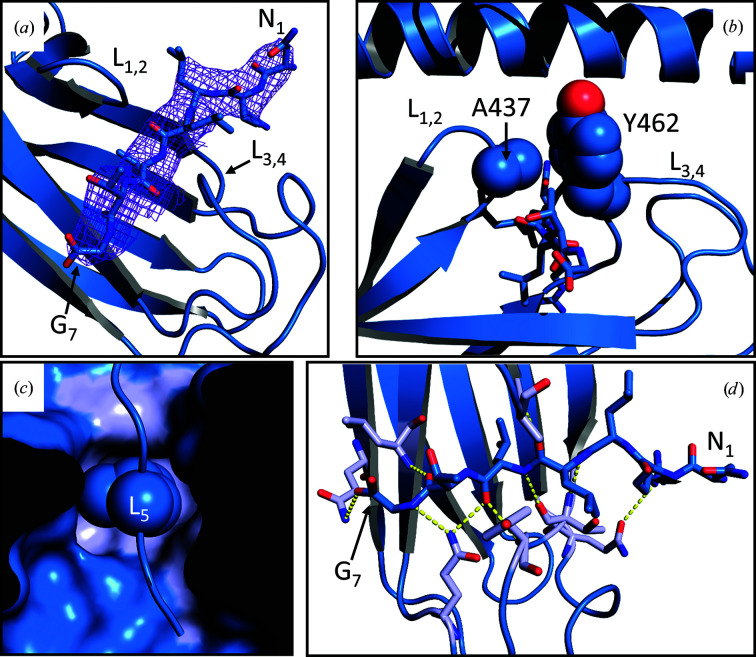
Substrate recognition by PfHsp70-x SBD. (*a*) Electron-density (2*F*_o_ − *F*_c_) OMIT map of the substrate peptide bound to PfHsp70-x SBD. The map was contoured at 1σ. The protein loops L_1,2_ and L_3,4_, which define the substrate-binding groove, are indicated. The substrate peptide, which is shown as a reference but was not included in the map calculations, is represented as sticks. (*b*) The Ala437–Tyr462 arch forming over the substrate-binding groove is shown. The side chains of the protein residues forming this arch are denoted as spheres. (*c*) Surface representation of the PfHsp70-x SBD hydrophobic cavity where the peptide L_5_ (side chain shown as spheres) docks. The surfaces of the protein residues that are directly involved in L_5_ binding are coloured light blue. (*d*) Hydrogen bonds inferred between the substrate peptide and PfHsp70-x SBD. Protein residues participating in hydrogen bonds are shown as sticks and coloured light blue. Hydrogen bonds are shown as dashed yellow lines.

The PfHsp70-x SBDβ harboured the substrate-binding site primarily between loops L_1,2_ and L_3,4_ (Fig. 2 ▸ *a*), which defined a hydrophobic groove. Residues Ala437 and Tyr462 of L_1,2_ and L_3,4_, respectively, formed an arch over this groove, thereby inhibiting substrate release (Fig. 2 ▸ *b*). All four copies of PfHsp70-x SBD in the asymmetric unit were complexed with a substrate peptide with sequence N_1_RLLLTG_7_; in three of the complexes all seven peptide residues could be resolved (Fig. 2 ▸ *a*), while only four residues (Leu_4_–Gly_7_) could be built for the last copy of the complex. Substrate binding was stabilized by the burial of Leu_5_ of the peptide in a hydrophobic pocket formed by Leu434, Phe459, Val469, Ile471, Ile505 and Val507 of SBDβ (Fig. 2 ▸ *c*), as well as additional hydrophobic interactions between Leu_4_ and Thr_6_ of the the peptide and the Ala437–Tyr462 arch of SBDβ (Fig. 2 ▸ *b*). Although the low resolution of this structure limits the accuracy with which hydrogen bonds can be inferred, we note that up to eight such bonds may form between the peptide Leu_3_–Gly_7_ backbone atoms and the SBDβ backbone (Ala437, Thr460, Tyr462 and Leu470) or side-chain (Gln466 and Gln 491) moieties (Fig. 2 ▸ *d*). Complex formation buried ∼567 Å^2^ of solvent-accessible surface area on average in the three PfHsp70-x SBD–substrate copies where all peptide residues could be resolved; thus, the complex interface accounts for nearly 50% of the total accessible peptide area. However, it should be noted that the NRLLLTG model peptide may not capture all of the interactions formed by the true substrates of the PfHsp70-x SBD.

### 3.2. Comparison of PfHsp70-x SBD with other Hsp70s   

Comparison of SBDs from Hsp70 homologues revealed a high level of amino-acid sequence identity (Supplementary Fig. S1), in particular for SBDβ. Crucially, the residues of the hydrophobic substrate-binding interface were nearly identical in human erythrocytic Hsp70s (Hsp70 and Hsc70; Bryk & Wiśniewski, 2017 ▸) and PfHsp70-x, and also in the more sequence-remote *E. coli* DnaK. However, Ala437 and Tyr462 of PfHsp70-x, which form the substrate arch in SBDβ, were identical in human chaperones, but their relative size was reversed (Met instead of Ala and Ala instead of Tyr) in DnaK. This reversal of amino-acid size in PfHsp70-x versus bacterial DnaK was previously recognized from the amino-acid sequence and may impact substrate specificity and release (Shonhai *et al.*, 2007 ▸; Zhu *et al.*, 1996 ▸). Further, the L_α,β_ loop was identical in the erythrocytic and *P. falciparum* chaperones, while it differed in the equivalent segment of DnaK by having larger amino acids (–TNDK– compared with –ASSG–) and being one residue longer. As opening and closing motions of the SBDβ/SBDα subdomains along the L_α,β_ loop have been shown to affect substrate binding and chaperone activity (Zhuravleva *et al.*, 2012 ▸; Kityk *et al.*, 2012 ▸), we concluded that PfHsp70-x SBD is likely to be functionally closer to its human erythrocytic counterparts than the prokaryotic chaperone.

**Figure 3:**
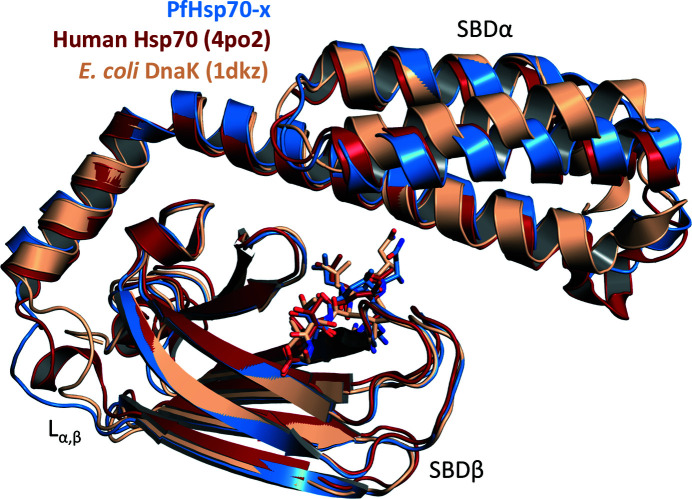
Comparison of PfHsp70-x SBD with homologues. Shown here is a structural superposition of PfHsp70-x SBD (blue) with the equivalent domains of human Hsp70 (red; PDB entry 4po2; Zhang *et al.*, 2014 ▸) and *E. coli* DnaK (wheat; PDB entry 1dkz; Zhu *et al.*, 1996 ▸). PfHsp70-x SBD is substantially similar to both of these domains; however, it is structurally closer to the human chaperone than to the prokaryotic counterpart.

The extent of the similarity of PfHsp70-x SBD to the human chaperones was underscored by structural comparisons. Superposition of the SBDs of human Hsp70 (PDB entry 4po2; Zhang *et al.*, 2014 ▸), DnaK (PDB entry 1dkz; Zhu *et al.*, 1996 ▸) and PfHsp70-x revealed broadly similar three-dimensional structures (Fig. 3 ▸). Nevertheless, alignment of the PfHsp70-x SBD with that of human Hsp70 showed a C^α^ r.m.s.d. of 2.45 Å on average (219 atoms aligned), whereas comparison of PfHsp70-x SBD with DnaK revealed a C^α^ r.m.s.d. of 3.03 Å (212 atoms aligned). Thus, the SBD of PfHsp70-x is structurally more similar to that of human Hsp70 than to *E. coli* DnaK. However, it is worth noting that the complexed state of Hsp70 SBD domains is generally structurally conserved, with an average C^α^ r.m.s.d. of only ∼1.3 Å seen in comparisons of DnaK, *E. coli* HscA (PDB entry 1u00; Cupp-Vickery *et al.*, 2004 ▸), human Hsp70 and bovine Hsc70 (PDB entry 3c7n; Schuermann *et al.*, 2008 ▸).

In conclusion, here we report the first crystallographic structure of the substrate-binding domain of PfHsp70-x. Analysis of the structure, and of the way in which a model substrate peptide was bound, suggested that PfHsp70-x adopts a canonical Hsp70-class chaperone SBD architecture. However, the PfHsp70-x SBD was more similar to those of human chaperones, such as those found in the erythrocyte, than to a prokaryotic counterpart. Although substrate selectivity depends on cross-talk between the Hsp70 SBD and ATP-binding domains (Swain *et al.*, 2007 ▸; Vogel *et al.*, 2006 ▸), which cannot be evaluated by the SBD structure alone, on the basis of similarities between the human Hsp70 and parasite Hsp70-x SBDs we propose that the parasite may be able to use human chaperones to supplement PfHsp70-x function, for example in maintaining the structure of parasite components exported to the host cell or for trafficking PfEMP1. Such a supplementary role of human Hsp70s may explain why the deletion or depletion of PfHsp70-x has only a partial impact on parasite survival and virulence (Charnaud *et al.*, 2017 ▸; Day *et al.*, 2019 ▸).

## Supplementary Material

PDB reference: Hsp70-x substrate-binding domain complexed with a hydrophobic peptide, 6zhi

Supplementary Figure S1. DOI: 10.1107/S2053230X2001208X/ow5024sup1.pdf

## Figures and Tables

**Table 1:** Macromolecule-production information

Source organism	*P. falciparum*
DNA source	Synthetic
Cloning vector	pFLOAT
Expression vector	pFLOAT
Expression host	*E. coli* Rosetta 2(DE3)
Complete amino-acid sequence of the construct produced	GPLLDVCPLSLGLETAGGVMTKLIERNTTIPTKKNQIFTTYADNQPGVLIQVYEGERAMTKDNNLLGKFQLEGIPPAPRSVPQIEVTFDIDANGILNVTALDKGTGKQNQITITNDKGRLSKDDIDRMVNDAEKYKEEDEQNKNRIEARNNLENYCYNVKNTLQDENLKTKIPKDDSEKCMKTVKSVLDWLEKNQTAETEEYNEKEKDISSVYNPIMTKIYQGASAQE

**Table 2:** Crystallization

Method	Sitting-drop vapour diffusion
Temperature (K)	293
Protein concentration	10 mg ml^−1^ protein + 5 m*M* NRLLLTG peptide
Buffer composition of protein solution	150 m*M* NaCl, 20 m*M* HEPES pH 7.2, 1 m*M* DTT
Composition of reservoir solution	0.1 *M* bicine/Trizma base pH 8.5, 10%(*w*/*v*) PEG 20 000, 20%(*w*/*v*) PEG MME 550, 0.03 *M* of each of diethylene glycol to pentaethylene glycol
Volume and ratio of drop	200 nl, 1:1 protein:mother liquor
Volume of reservoir (µl)	40

**Table 3:** Data collection and processing Values in parentheses are for the outer shell.

Diffraction source	Beamline I03, Diamond Light Source
Wavelength (Å)	0.9793
Temperature (K)	100
Detector	PILATUS3 6M, Dectris
Rotation range per image (°)	0.1
Total rotation range (°)	360
Exposure time per image (s)	0.01
Space group	*P*2_1_
*a*, *b*, *c* (Å)	71.07, 93.85, 85.30
α, β, γ (°)	90, 99.47, 90
Resolution range (Å)	93.85–3.25 (3.31–3.25)
Total No. of reflections	120016 (5297)
No. of unique reflections	17457 (796)
Completeness (%)	99.8 (94.1)
Multiplicity	6.9 (6.7)
〈*I*/σ(*I*)〉	8.7 (1.5)
*R*_p.i.m._	0.048 (0.777)
CC_1/2_	0.997 (0.568)
Overall *B* factor from Wilson plot (Å^2^)	94.35

**Table 4:** Structure refinement Values in parentheses are for the outer shell.

Resolution range (Å)	84.13–3.25 (3.28–3.25)
Completeness (%)	99.7
σ Cutoff	*F* > 0.000σ(*F*)
No. of reflections, working set	17437 (399)
No. of reflections, test set	843
Final *R*_cryst_	0.297 (0.289)
Final *R*_free_	0.321 (0.299)
No. of non-H atoms	
Protein	7012
Ligand	0
Water	0
R.m.s. deviations	
Bonds (Å)	0.009
Angles (°)	1.12
Average *B* factors (Å^2^)	152.5
Protein geometry	
Ramachandran favoured (%)	99.54
Ramachandran disallowed (%)	0.00
Favoured rotamers (%)	99.5
Poor rotamers (%)	0.00
*MolProbity* clashscore, all atoms	15.1
*MolProbity* clashscore, percentile	97th
*MolProbity* score	1.68
*MolProbity* score, percentile	100th
