# Supplementary material for: Structure of the substrate-binding domain of Plasmodium falciparum heat-shock protein 70-x
Source: Acta Crystallogr F Struct Biol Commun. 2020 Sep 28;76(Pt 10):495–500. doi: 10.1107/S2053230X2001208X (PMC7531245; doi:10.1107/S2053230X2001208X)
Supplement: Supplementary file 1 [file f-76-00495-sup1.pdf]

STRUCTURAL BIOLOGY COMMUNICATIONS

Volume 76 (2020)

Supporting information for article:

Structure of the *Plasmodium falciparum* heat-shock protein 70-x substrate-binding domain

Julia Schmidt and Ioannis Vakonakis

Figure S1

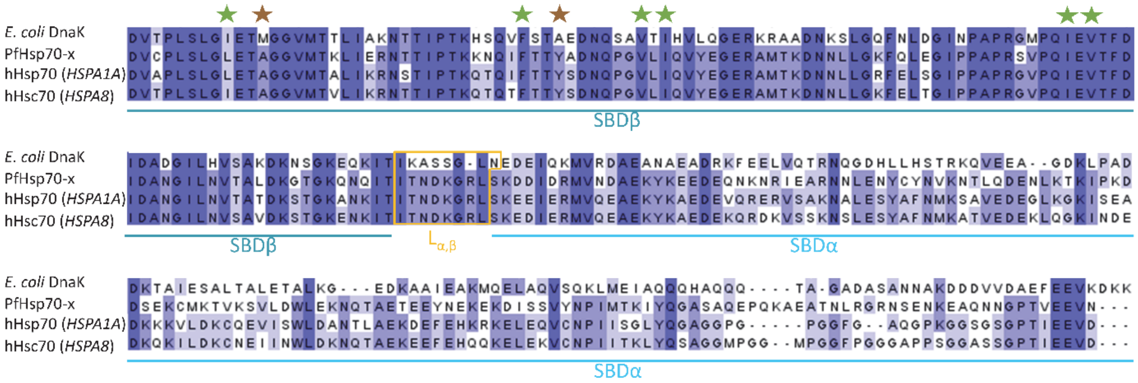

Sequence alignment of PfHsp70-x SBD with homologous domains. Shown here is a sequence alignment of PfHsp70-x SBD (UniProt K7NTP5, residues 426-679) against the equivalent domains of *E. coli* DnaK (UniProt P0A6Y8, residues 393-638) and of human erythrocytic Hsp70-class chaperones (Hsp70, also known as HSPA1A, UniProt P0DMV8, residues 395-641; Hsc70, also known as HSPA8, UniProt P11142, residues 395-646). The sequences of SDBβ and SDBα sub-domains are underlined, the $L_{\alpha,\beta}$ loop connecting the two sub-domains is boxed in yellow. Green stars above the sequence denote SBDβ residues forming the hydrophobic pocket that binds the substrate L_5 amino acid. Brown stars denote SDBβ residues forming an arch above the substrate-binding groove.
